# Sex Differences in the Response to Different Tinnitus Treatment

**DOI:** 10.3389/fnins.2020.00422

**Published:** 2020-05-12

**Authors:** Annemarie Van der Wal, Tine Luyten, Emilie Cardon, Laure Jacquemin, Olivier M. Vanderveken, Vedat Topsakal, Paul Van de Heyning, Willem De Hertogh, Nancy Van Looveren, Vincent Van Rompaey, Sarah Michiels, Annick Gilles

**Affiliations:** ^1^Department of Otorhinolaryngology – Head and Neck Surgery, Antwerp University Hospital, Edegem, Belgium; ^2^Department of Rehabilitation Sciences and Physiotherapy, Antwerp University, Antwerp, Belgium; ^3^Department of Translational Neurosciences, Antwerp University, Antwerp, Belgium; ^4^Hoorzorg Van Looveren BVBA, Borsbeek, Belgium; ^5^Department of Education, Health and Social Work, University College Ghent, Ghent, Belgium

**Keywords:** gender, tinnitus, gender-related outcome, TRT, CBT, neuromodulation, HDtDCS, tDCS

## Abstract

**Introduction:**

Tinnitus is a complex symptom requiring a thorough multidisciplinary assessment to construct an individual’s tinnitus profile. The Antwerp University Hospital hosts a tertiary tinnitus clinic providing intensive, multidisciplinary tinnitus care in the form of combinational psychological treatment with either Tinnitus Retraining Therapy (TRT)/Cognitive Behavioral Therapy (CBT) or TRT/eye movement desensitization and reprocessing therapy (EMDR), high-definition transcranial direct current stimulation (HD-tDCS), and physical therapy treatment (in cases of somatic influence of the neck or the temporomandibular area). Several factors may contribute to therapy effect of which the role of gender has recently gained more interest. As such, the current manuscript explores gender differences in the outcome of different tinnitus treatments.

**Methods:**

Data on treatment outcome of four distinct tinnitus treatments (1. HD-tDCS; 2. orofacial physical therapy; 3. combination TRT + CBT; and 4. combination TRT + EMDR) were pooled and compared. Treatment outcome was assessed via the Tinnitus Functional Index (TFI). Participants completed the TFI at baseline, immediately after treatment and after 9 weeks (±3 weeks) follow-up. To explore the effect of gender on different treatment outcomes, a linear mixed model was designed including *Time point*, *Gender*, and *Therapy Group* as fixed factors as well as all interactions between these factors.

**Results:**

TFI scores improved significantly over time regardless of therapy group (*p* < 0.0001). A mean TFI decrease of at least 13 points was obtained by all participants except by those in the HD-tDCS. Significant interactions between Gender and Time point were identified in all groups except for the TRT +EMDR group. Female subjects improved more extensively than males in the HD-tDCS (*p* = 0.0009) and orofacial therapy group (*p* = 0.0299). Contrarily, in the TRT +CBT group, male participants showed a significant improvement whereas the mean TFI scores of female subjects remained on baseline levels (*p* = 0.0138).

**Conclusion:**

Our data suggest that male and female tinnitus patients seem to react differently to different therapy options. We strongly encourage further prospective studies to discern the relevance of gender in therapy outcome.

## Introduction

Tinnitus, the perception of sound in the absence of an external sound source, is a frequently experienced symptom in modern society. The prevalence of tinnitus in an adult population is around 15% (Seidman and Jacobson, 1996; Gilles et al., 2012). In 2–3% of patients, the tinnitus is sufficiently bothersome to affect the quality of life due to the association with anxiety, depression, sleep disorders, concentration difficulties, and elevated stress levels (Henry et al., 2005). As a consequence, many patients find their way to the clinic seeking for alleviating treatment. In the management of tinnitus, a multi-disciplinary approach is essential in order to tailor therapy toward the patient’s requirements and needs. Thorough evaluation of the patient comprises a systematic history including tinnitus characteristics, potential tinnitus triggers, and the evaluation of coexisting symptoms such as subjective hearing loss, otalgia, decreased speech understanding (Gilles et al., 2016), vertigo, and hyperacusis (Van de Heyning et al., 2015). The presence of cervical spine dysfunction as well as bruxism or known history of temporomandibular dysfunctions should be inquired and assessed accordingly as somatosensory influences may cause tinnitus and/or increase pre-existing tinnitus loudness or alter pitch (Michiels et al., 2015a, 2018b). In addition, the comorbidity with depression or anxiety disorders should be assessed, as emotional factors typical in these disorders are strong predictors of poor adjustment to the symptom of tinnitus (Zoger et al., 2006; Pattyn et al., 2016). Following extensive anamnesis, further multidisciplinary investigations as well as imaging may be required leading toward an individual tinnitus profile guiding the patient toward the most appropriate patient-specific therapy (Van de Heyning et al., 2015).

Yearly, approximately 1500 patients consult the tertiary expertise tinnitus clinic [Tinnitus Treatment and Research Centre Antwerp (TINTRA)] at the Antwerp University Hospital, with tinnitus being their primary complaint. Through a multidisciplinary approach, patients receive treatment according to their tinnitus profile, underlying causes/mediators and psychological burden. For all patients, pre-therapeutic tinnitus burden is measured at baseline, post-therapy, and at 9 weeks (±3 weeks) follow-up moment using tinnitus questionnaires.

Several treatments provided at TINTRA are psychology-based focusing on altering the coping strategies in order to change emotional responses toward the tinnitus. Cognitive Behavioral Therapy (CBT) in particular aims to change non-constructive cognitive distortions/behaviors and develop personal coping strategies targeting the tinnitus issues. Tinnitus Retraining Therapy (TRT) on the other hand is a habituation therapy in which directive counseling aims to reclassify the tinnitus percept to a neutral signal in combination with the use of sound therapy. Both CBT and TRT have proven to be effective in the treatment of tinnitus (Jastreboff and Jastreboff, 2000; Jastreboff, 2007, 2015; Cima et al., 2014). Eye movement desensitization and reprocessing (EMDR) therapy is a form of psychotherapy in which the patient is enquired to recall distressing thoughts/images (i.e., associated with the tinnitus) after which the therapist directs the patient with bilateral sensory input (i.e., hand tapping, auditory stimuli, or side-to-side eye movements). EMDR is a widely used technique in the treatment of post-traumatic stress disorder (Guideline Development Panel for the Treatment of PTSD in Adults, and American Psychological Association, 2019). In the field of tinnitus, a first preliminary study on EMDR was recently published showing promising results as significant improvement on tinnitus burden (measured by the Tinnitus Handicap Inventory) and depressive symptoms (measured by the Beck Depression Inventory) was shown up until 6 months after EMDR treatment (Phillips et al., 2019).

Also, transcranial direct current stimulation (tDCS) has been a topic of research at TINTRA. TDCS is a form of neuromodulation delivering a constant, low direct current to the brain through electrodes positioned on the head. Up until now, a total of 31 studies evaluated the effects of tDCS on tinnitus reporting various degrees of effect, ranging from no effect to significant tinnitus reduction. A large heterogeneity in used tDCS protocols and outcomes is apparent which constrains the comparability of these studies (Lefaucheur, 2016; Lefaucheur et al., 2017). At our department tDCS trials have shown clinically significant improvement in 32% of patients with large inter-individual variability (Jacquemin et al., 2018).

For patients who experience a somatosensory influence on tinnitus originating in a dysfunction of the cervical spine or temporomandibular area (Michiels et al., 2015b, 2016b, 2018a), physiotherapy treatment is provided. Depending on the area of the dysfunction that is primarily influencing the tinnitus, the physical therapy treatment is adjusted. In case of a primary influence from the cervical spine, a multimodal manual physical therapy can be provided (Michiels et al., 2016a, 2017). This type of treatment showed a significant improvement on the global perceived effect in 53% of the patients in our clinic (Michiels et al., 2016c). In case temporomandibular dysfunctions are primarily influencing the tinnitus, orofacial physiotherapy is provided, when needed combined with occlusal splints provided by the dentist. A recent systematic review on the topic showed promising results, although differences in treatment modalities and outcome measures make it hard to draw any definitive conclusions (Michiels et al., 2019).

As the tinnitus population is highly heterogeneous, a one-therapy-fits-all approach is non-existent and many treatments may add to tinnitus alleviation depending on the patients’ tinnitus profile. Due to the heterogeneity, treatment outcomes also tend to vary tremendously. Recently the role of gender on the perception of tinnitus has gathered more attention. In a study by Seydel et al. (2013), women showed more tinnitus-related distress compared to men with this effect depending on age and duration of the tinnitus. These results were partially confirmed by a recent study by Han et al. (2019) and some authors have mentioned gender difference in the amount of tinnitus-related distress as well (Hiller and Goebel, 2006), whereas others did not find any gender differences (Meric et al., 1998; Pinto et al., 2010). As a result, the role of gender remains elusive. Recently, severe tinnitus was shown to be associated with an increased risk of suicide attempts in female patients only (Lugo et al., 2019). Interestingly, those patients who had been diagnosed with tinnitus in a clinical setting were no longer at risk, highlighting the need for specialty care in the tinnitus population. If males and females perceive tinnitus differently and, as a consequence, possibly show distinct reactions toward therapeutic intervention, caregivers should take these gender differences into account. The current manuscript reports on the effects of gender on the outcomes of the distinct tinnitus therapies provided at TINTRA.

## Materials and Methods

### Study Protocol

The current manuscript describes the effects of gender on tinnitus treatment outcome. Therefore, all data collected prior to treatment, post-treatment, and at a follow-up visit (9 ± 3 weeks) of patients who received tinnitus treatment, were analyzed retrospectively. All patients had chronic, non-pulsatile, subjective tinnitus for longer than 3 months. Patients with active middle ear pathology were excluded from analysis. Pure tone audiometry was performed at baseline using a two-channel Interacoustics AC-40 audiometer and headphones in a soundproof booth. Air conduction thresholds were measured at 125, 250, 500, 1000, 2000, 3000, 4000, 6000, and 8000 Hz. Bone conduction thresholds were tested at 250, 500, 1000, 2000, 3000, and 4000 Hz. Tinnitus evaluation was performed using self-administering questionnaires.

### Treatment

#### Psychological Treatment

Two combination psychological therapies were provided with either TRT +CBT combination (five sessions TRT/five sessions CBT) or TRT+EMDR combination (five sessions TRT/five sessions EDMR). All therapies were provided by licensed TRT audiologists and CBT/EMDR psychologists. The full approach of this bimodal psychotherapy is described in Luyten et al. (2019).

#### High-Definition Transcranial Direct Current Stimulation

Patients received six sessions of high-definition tDCS (HD-tDCS) over 3 weeks’ time (2x/week) with silver/silverchloride electrodes placed on the right dorsolateral prefrontal cortex (dLPFC). A direct current of 2 mA was applied with a 20 s fade-in/fade-out time delivered by a battery-driven 1 × 1 tDCS low-intensity stimulator and 4 × 1 multichannel stimulation adaptor (Soterix Medical Inc., New York, NY, United States) as described in the HD-tDCS stimulation guidelines (Villamar et al., 2013). During each session, the patient received 20 min of stimulation.

#### Conservative Temporomandibular Treatment

Patients received a maximum of 18 sessions of orofacial therapy during a fixed time window of 9 weeks. This therapy was primarily directed to the temporomandibular joint and masticatory muscles. If present, cervical spine dysfunctions were treated as well, using a combination of manual mobilizations and exercise therapy. In case of severe bruxism, an occlusal splint was provided in addition to the physiotherapy. Therapists, providing the orofacial physiotherapy, were trained to the study protocol prior to the start of the study. More details can be found in the published study protocol (Michiels et al., 2018b).

#### Treatment Allocation

Treatment allocation to one of the four interventions described below was based on patients’ complaints and needs as discussed during an intake visit at the TINTRA tinnitus consultation. For instance, only patients with self-reported temporomandibular complaints were considered for the orofacial therapy. Different therapy options were discussed based on individual patients’ tinnitus profiles, and final treatment decisions were made by the patients. Thus, a certain level of selection bias was present in the treatment allocation, but this was solely based on individual patients’ needs. Demographic variables, such as gender, were never taken into account as a deciding factor for treatment choice.

### Ethics

All patients filled out an informed consent in which permission was granted to use their data gathered during and prior to tinnitus treatment. Ethics Committee approval numbers involved in the current analysis are 16/41/415, 16/35/360, and 16/48/513.

### Questionnaires

#### Tinnitus Functional Index

The Tinnitus Functional Index (TFI) (Meikle et al., 2012; Rabau et al., 2014) consists of 25 items assessing the tinnitus severity as well as the impact of tinnitus in daily life. In addition to a total score reflecting the total tinnitus burden, eight subscales define the level of inconvenience for the following aspects: intrusiveness, reduced sense of control, cognitive interference, sleep disturbance, auditory difficulties, interference with relaxation, reduced quality of life, and emotional distress. The TFI has proven to be useful in the assessment of treatment-related changes in tinnitus. As such, a reduction of 13 points on the total TFI score after tinnitus treatment is considered as a clinically relevant and subjectively perceived reduction for the patient (Meikle et al., 2012).

#### Visual Analog Scale

The mean tinnitus loudness throughout the day was assessed by use of a Visual Analog Scale (VAS). In this case, the patient had to indicate the mean tinnitus loudness over the last week on a scale from 0 (no tinnitus at all) to 100 (the most extreme loudness one can imagine) by use of a ruler.

#### Hyperacusis Questionnaire

The Hyperacusis Questionnaire (HQ) (Khalfa et al., 2002; Meeus et al., 2010) determines the presence/absence of hyperacusis. According to Khalfa’s original HQ, one can speak of hyperacusis when the score on the HQ is 28 or higher (Khalfa et al., 2002).

#### Hospital Anxiety and Depression Scale

The Hospital Anxiety and Depression Scale (HADS) is a screening tool to detect symptoms of anxiety and/or depression (Zigmond and Snaith, 1983). The HADS consists of 14 items in total assessing increased signs of anxiety (seven items) or depression (seven items). For each of the subscales, a score between 8 and 10 is considered as “borderline” while a score of 11 and higher is considered as “case.”

### Statistics

A linear mixed model was designed using JMP^®^ Pro software (JMP^®^ Pro, Version 14.0.0, 2018 SAS Institute Inc., Cary, NC, United States, 1989–2019) to explore the effects of gender on outcomes of different tinnitus treatments. Total TFI score was chosen as the outcome variable. The model applied the following fixed factors: Time point (baseline, post-treatment, follow-up), Gender (male, female), and Therapy group (HD-tDCS, orofacial therapy, TRT combined with CBT, TRT combined with EMDR). All possible two-way interactions between these factors were added to the model, as well as the three-way interaction Time point^∗^Gender^∗^Therapy group. Possible confounding factors were added to the model in a stepwise additive manner. These factors were age, hearing level (pure tone averages for 1, 2, and 4 kHz), tinnitus characteristics (type, duration, and laterality of the tinnitus), scores on the HADS depression and anxiety subscales, and scores on the HQ. Considerably more men than women were in all therapy groups except for the orofacial therapy group, in which the gender distribution was more balanced. Participant was added as a random intercept. *Post hoc* analyses were performed using linear mixed models for each of the four therapy groups separately. Here, Time point and Gender were added as fixed factors with an additional two-way interaction of Time point^∗^Gender. *P*-values of < 0.05 were considered significant.

## Results

Data from 316 patients were included in the analysis. An overview of patients’ characteristics at baseline can be found in Table 1. Considerably more men than women were included in all therapy groups except for the orofacial therapy group, in which the gender distribution was more balanced. Age varied slightly between therapy groups, with mean age in the HD-tDCS group being the highest and participants in the orofacial therapy group being the youngest. Hearing levels did not differ significantly between groups, nor did tinnitus characteristics (duration, type, and laterality of the tinnitus) or TFI scores at baseline. Some group level differences were found for the additional questionnaire scores, with participants in the HD-tDCS and orofacial therapy groups scoring lower on the anxiety subscale of the HADS and on the HQ than patients in both psychotherapeutic groups.

**TABLE 1:** Demographic details and tinnitus characteristics at baseline.

	**HD-tDCS**			**Orofacial therapy**			**TRT + CBT**			**TRT + EDMR**		
	**(*n* = 117)**			**(*n* = 109)**			**(*n* = 44)**			**(n = 46)**		
	**M**	**F**	***p***	**M**	**F**	***p***	**M**	**F**	***p***	**M**	**F**	***p***
**Gender** (*n*)	97	20		56	53		34	10		30	16	
**Age** (years: mean [SD])	50.02 [12.60]	53.65 [10.81]	0.2329	42.68 [15.44]	47.68 [14.22]	0.0819	49.32 [13.46]	44.5 [12.34]	0.3165	48.33 [12.44]	46 [12.13]	0.5443
**Hearing level: PTA** (db HL: mean [SD])	19.29 [13.48]	15.38 [11.84]	0.2309	13.85 [13.02]	16.30 [13.92]	0.3491	19.75 [15.60]	8.92 [5.93]	0.0385	17.22 [14.41]	16.09 [17.39]	0.8151
**Tinnitus characteristics**												
Tinnitus duration (years: mean[SD])	6.59 [7.49]	4.06 [5.63]	0.1546	5.27 [7.30]	4.98 [7.14]	0.8351	9.08 [10.56]	3.35 [1.81]	0.0978	8.23 [8.98]	3.97 [2.89]	0.0728
Tinnitus laterality (*n*: right/left/bilateral/central)	13/12/57/15	1/3/13/3	0.7066	6/9/27/13	6/5/33/7	0.2132	2/8/15/9	0/3/6/1	0.4497	3 / 6 / 12 / 9	2 / 4 / 6 / 4	0.9624
Tinnitus type (*n*: pure tone/noise/polyphonic)	67/22/8	12/4/4	0.3468	32/16/7	22/25/4	0.0862	25/7/2	3/4/3	0.0310	18 / 8 / 4	11 / 3 / 2	0.8126
TFI scores (mean [SD])	45.5 [18.62]	55.5 [23.44]	0.0390	50.64 [18.67]	54.02 [15.35]	0.3050	48.92 [22.10]	54.24 [22.11]	0.5068	51.37 [18.31]	58.58 [18.22]	0.2098
**Questionnaire scores**												
HADS anxiety (mean [SD])	7.29 [3.60]	9.2 [4.82]	0.0443	8.91 [4.06]	8.43 [4.22]	0.5524	8.79 [4.42]	12.6 [4.43]	0.0212	8.53 [4.01]	11 [3.92]	0.0512
HADS depression (mean [SD])	6.77 [4.05]	7.65 [5.49]	0.4106	7.09 [4.49]	5.36 [4.54]	0.0488	6.91 [4.25]	9.7 [4.52]	0.0794	7.53 [4.61]	7.56 [4.07]	0.9831
HQ scores (mean [SD])	18.34 [7.74]	21.25 [9.59]	0.1448	17.22 [7.19]	17.61 [8.89]	0.8039	20.74 [9.15]	26.9 [7.72]	0.0600	20.93 [7.90]	26.81 [3.87]	0.0077

*HD-tDCS: High-Definition transcranial Direct Current Stimulation; TRT: Tinnitus Retraining Therapy; CBT: Cognitive Behavioral Therapy; EMDR: eye movement desensitization and reprocessing therapy; PTA: Pure Tone Average for 1, 2, and 4 kHz; TFI: Tinnitus Functional Index; HADS: Hospital Anxiety and Depression Scale; HQ: Hyperacusis Questionnaire. Standard least squares (for continuous variables) or nominal logistic models (for dichotomous variables) were designed to test whether male and female participants in each of the four therapy groups differed significantly; resulting *p-*values are shown in the last column.*

**FIGURE 1:**
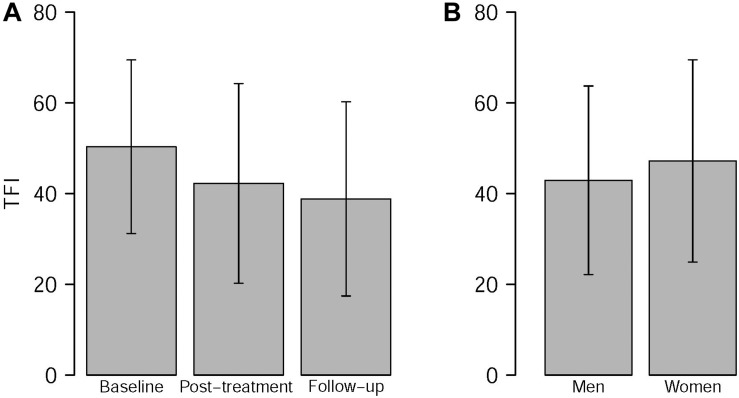
Main effects of time point **(A)** and gender **(B)**. **(A)** TFI scores change significantly over time, independently of therapy group or gender (*p* < 0.0001). TFI scores at baseline: 50.34 ± 19.13; TFI scores at the post-treatment time point: 42.24 ± 21.99; TFI scores at follow-up: 38.84 ± 21.39; mean ± SD. **(B)** TFI scores are higher in women than in men, independently of therapy group or time point (*p* = 0.0496). TFI scores for women: 47.19 ± 22.28; TFI scores for men: 42.93 ± 20.79; mean ± SD. Error bars represent SD. TFI: Tinnitus Functional Index.

**FIGURE 2:**
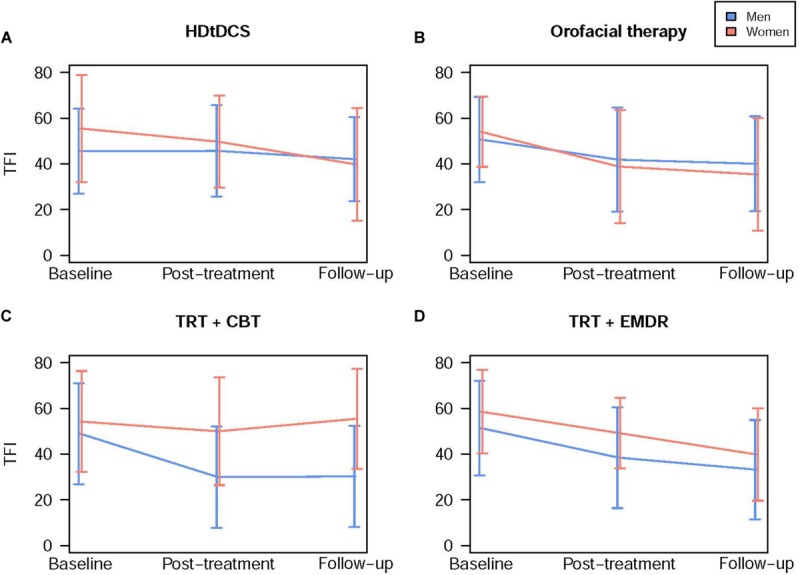
Treatment outcome in different therapy groups differs according to gender. Blue lines represent male subjects and red lines represent female subjects. Data are presented as mean TFI scores for each time point ± SD. **(A)** Female participants benefit more from HDtDCS treatment than men. TFI scores decreased more in female participants (−15.83 ± 24.27) than male (−2.53 ± 13.79) from baseline to follow-up. **(B)** Female participants benefit more from orofacial therapy than men. TFI scores decreased more in female participants (−19.02 ± 16.85) than male (−10.53 ± 19.65) from baseline to follow-up. **(C)** Male participants benefit more from TRT combined with CBT than women. TFI scores decreased more in male participants (−22.42 ± 23.02) than female (0.44 ± 21.26) from baseline to follow-up. **(D)** Male and female participants benefit equally from TRT combined with EMDR. TFI scores decreased similarly in male (−19.1 ± 15.82) and female participants (−16.46 ± 14.3) from baseline to follow-up. TFI: Tinnitus Functional Index; HDtDCS: High-Definition transcranial Direct Current Stimulation; TRT: Tinnitus Retraining Therapy; CBT: Cognitive Behavioral Therapy; EMDR: Eye Movement Desensitization and Reprocessing.

Results of the linear mixed model analysis are summarized in Table 2. Of all putative confounding factors, only the HADS depression subscale score and the HQ score were found to contribute significantly to the model. All other possible confounding factors (age, hearing level, tinnitus characteristics, and HADS anxiety subscale scores) did not have any demonstrable effect on the model and were excluded from the analysis. Thus, the final model included two additional factors (HADS depression scores and HQ scores) next to the fixed factors Time point, Gender, and Therapy group. Overall, a significant fixed effect of time point was found, with TFI scores decreasing over time for all therapy groups and for both genders (*p* < 0.0001) (Figure 1A). Additionally, the main effect of gender was found to be significant, with women generally having a higher TFI score than men (*p* = 0.0496) (Figure 1B). A significant interaction between Time point and Therapy group was identified (*p* = 0.0023), indicating that TFI scores of participants in different therapy groups evolved differently over time. Crucially, a significant three-way interaction between Time point, Gender, and Therapy group was found (*p* = 0.0002). This interaction implies that treatment response in the different therapy groups was modulated by gender (Figure 2).

**TABLE 2:** Results of the linear mixed model analysis.

	***p-*value**
**Fixed factors**	
Time point	< 0.0001
Gender	0.0496
Therapy group	0.1072
HADS depression subscale scores	< 0.0001
HQ scores	0.0007
**Interactions**	
Time point ^∗^ Gender	0.7613
Time point ^∗^ Therapy group	0.0023
Gender ^∗^ Therapy group	0.5761
Time point ^∗^ Gender ^∗^ Therapy group	0.0002

*HADS: Hospital Anxiety and Depression Scale; HQ: Hyperacusis Questionnaire.*

To further explore this highly significant three-way interaction, *post hoc* analyses were performed for each therapy group. Similar to the primary analysis, HADS depression subscale scores and HQ scores were added to the model as confounding factors. In three out of four therapy groups, significant interactions between *Time point* and *Gender* were found. These interactions indicate that the evolution of TFI scores depended on gender in these specific therapy groups. In the HD-tDCS (*Time point^∗^Gender*: *p* = 0.0009) and orofacial therapy group (*Time point^∗^Gender*: *p* = 0.0299), female participants generally had higher TFI scores at baseline, but improved more extensively than male subjects (Figures 2A,B). On average, TFI scores of female participants in the HD-tDCS group decreased by 15.83 ± 24.27 points from baseline to follow-up, whereas male participants’ TFI scores dropped by only 2.53 ± 13.79 points. In the orofacial therapy group, female participants’ TFI scores decreased by 19.02 ± 16.85 points while scores of male participants decreased by 10.53 ± 19.65 points.

Contrarily, in the TRT + CBT group, male subjects improved significantly over time whereas female participants’ mean TFI scores only decreased slightly after treatment (*p* = 0.327) but did not remain stable at follow-up (Figure 2C) (*Time point^∗^Gender*: *p* = 0.0138). From baseline to follow-up, male participants’ TFI scores decreased by 22.42 ± 23.02 points whereas TFI scores of female subjects in this group actually increased marginally with 0.44 ± 21.26 points. Finally, in the TRT + EMDR group, the two-way interaction *Time point^∗^Gender* was not significant (*p* = 0.5240), with men and women improving over time in a highly similar way (Figure 2D). The decrease in TFI scores from baseline to follow-up was 19.1 ± 18.52 and 16.46 ± 14.3 points in female and male participants, respectively.

HADS depression subscale scores and HQ scores were found to contribute significantly to the model. *Post hoc* analyses revealed that both questionnaire scores had a significant effect on tinnitus severity, with higher scores on both questionnaires equaling higher total TFI scores. These effects were not dependent on either *Time point* or *Gender*, as evidenced by the non-significance of the interactions between these questionnaire scores and *Time point* (*p* = 0.5914 for HADS depression scores, *p* = 0.1814 for HQ scores) or *Gender* (*p* = 0.6806 for HADS depression scores, *p* = 0.1868 for HQ scores).

## Discussion

The large variability in tinnitus treatment outcomes may be driven by differences in individual patient characteristics. The current manuscript retrospectively explored whether gender may account for any of this heterogeneity. We report remarkable gender effects on treatment outcomes of several tinnitus therapy options. Overall, our results indicate that gender might be an influential mediator of treatment outcome.

In a large group of tinnitus patients, we found that women benefited from orofacial physiotherapy to a greater extent than men. In the general population, a recent meta-analysis showed that the risk for developing temporomandibular disorders (TMD) is twice as high in women than in men (Bueno et al., 2018). Likewise, a study of Vielsmeier et al. (2011) showed that significantly more women than men had TMD in a population of patients with tinnitus, something which was also found in the general population (Edvall et al., 2019). Therefore, we hypothesize that the proportion of true TMD sufferers was higher in female than in male patients. This gender difference in baseline TMD burden may then explain the higher success ratio of conservative orofacial treatment in female tinnitus patients. Currently, no other studies reported on gender differences in orofacial treatment effect on tinnitus complaints. It must also be noted that although statistically significant, the difference in decrease in TFI score between males and females in this treatment group is small. Our results should therefore be confirmed in future research, specifically designed to investigate gender differences.

In our study population, women also demonstrated a better treatment response after receiving consecutive sessions of HD-tDCS. These results are in agreement with Frank et al. (2012), who reported larger beneficial effects of frontal tDCS in women. Both protocols comprised anodal stimulation of the right dLPFC, suggesting that this gender difference might be driven by true underlying physiological differences. Indeed, dLPFC volume has been shown to be greater in women than men (Schlaepfer et al., 1995; Luders et al., 2009), and anodal tDCS of the prefrontal cortex might have greater effects in females (Fumagalli et al., 2010). These findings cannot be explained by the amount of current intensity going through the brain as it was previously shown that females receive significantly less current compared to males when targeting parietal and frontal areas due to more dense parietal bone in females (Russell et al., 2014). A significant difference in TFI at baseline between males and females was observed with females showing higher TFI scores which may imply a greater opportunity for improvement in this population. However, even after correcting for this difference in baseline TFI score, the effect of gender over time remained significant. In addition, no equal proportion of male and female subjects was obtained in this therapy group so these results need to be interpreted with caution.

Conversely, in our study population, male patients benefited more than females from TRT combined with CBT, while both males and females experienced significant improvement through EMDR therapy. Although some evidence exists for gender differences in tinnitus perception and distress, literature on gender effects on the efficacy of psychological tinnitus treatments is scarce. To date, meta-analyses on psychotherapy outcomes report minimal to no gender differences (Bowman et al., 2001; Felmingham and Bryant, 2012; Swift et al., 2013; Tao et al., 2015; Budge and Moradi, 2019).

In a study reporting on the long-term effects of TRT combined with cognitive-behavioral elements, Seydel et al. (2010) reported subtle gender differences on treatment outcome. Male and female patients showed similar outcomes of this treatment overall, but women experiencing longer (>2 years and especially >10 years) tinnitus duration were less likely to maintain positive treatment effect at a 1-year follow-up time point. In our dataset, we did not identify any effects of tinnitus duration. However, the relatively long average tinnitus duration (7.78 years) might account for this gender difference on the outcome of a treatment combining TRT and CBT. About 70% of the female participants included in the TRT + CBT group reported a psychiatric diagnosis in the present or the past. These findings suggest that 10 sessions might not be sufficient to alleviate the complex tangle of aggravating complaints. The proportion of patients with psychiatric comorbidity was lower in the TRT + EMDR group. Given the fact that 40% of female participants did report a significant decrease of 13 points or more in the TRT + CBT group, we emphasize the identifiable influence of psychiatric comorbidity on treatment outcome. However, the cognitive behavioral approach could have influenced therapy outcome on the basis of the cognitive therapeutic techniques that were used. Some evidence can be found on women to experience more therapeutic gain through an emotion-focused treatment (Ogrodniczuk et al., 2001; Matud, 2004; Ogrodniczuk, 2006; Meléndez et al., 2012) whereas men tend to react better to a problem-focused therapy which is more integrated in the CBT approach (Samoilov and Goldfried, 2000). More empirical evidence is required to investigate whether these findings can be replicated. We interpret these results with caution, taking into account the importance of the individual characteristics of the female participants in this considerably small sample size compared to the proportion of male participants.

The results presented here are highly explorative, and large prospective trials are needed to confirm or disprove the gender effects we demonstrate. It would be especially prudent for future studies to ensure an even inclusion of men and women, as the gender balance is disturbed in many studies, as well as in several of the therapy groups discussed in this paper. Furthermore, as we identified additional effects of symptoms of depression and hyperacusis, controlling for these factors is undeniably crucial.

We have demonstrated considerable effects of gender on tinnitus treatment outcome for different therapy options. Our results suggest that women might experience greater effects of orofacial physiotherapy and transcranial direct current stimulation compared to men. Within the psychotherapeutic treatments, we identified subtle gender differences between the outcome of CBT compared to eye movement desensitization and retraining, but mainly note that individual differences and psychiatric comorbidity affect the therapeutic pathway and treatment outcome. We report remarkable gender effects on treatment outcomes of several tinnitus therapy options. Overall, our results indicate that gender might be of influence for treatment outcome. Consequently, it might be important to consider gender when estimating the chance for treatment success.

## Data Availability Statement

The datasets generated for this study are available on request to the corresponding author.

## Ethics Statement

The studies involving human participants were reviewed and approved by the Ethical committee of the University Hospital Antwerp. The patients/participants provided their written informed consent to participate in this study.

## Author Contributions

AV, TL, LJ, and NV contributed to data collection. AG and SM contributed to PI. VV, OV, VT, PV, and WD contributed to protocol and statistics. EC, SM, and AG contributed to analysis. AG, AV, TL, and EC contributed to manuscript writing.

## Conflict of Interest

The authors declare that the research was conducted in the absence of any commercial or financial relationships that could be construed as a potential conflict of interest.
